# Reopening safely – Lessons from Taiwan’s COVID-19 response

**DOI:** 10.7189/jogh.10.020318

**Published:** 2020-12

**Authors:** Cheryl Lin, Jewel Mullen, Wendy E Braund, Pikuei Tu, John Auerbach

**Affiliations:** 1 Policy and Organizational Management Program, Duke University, Durham, North Carolina, USA; 2 Dell Medical School, University of Texas at Austin, Austin, Texas, USA; 3 University of Pittsburgh Graduate School of Public Health, Pittsburgh, Pennsylvania, USA; 4 Trust for America's Health, Washington, DC, USA

As many states and nations wrestle with resurgence of COVID-19, Taiwan has observed 46 days without locally transmitted cases at a 0.61% test-positivity rate and 0.3/million mortality rate, among the lowest globally (441 confirmed cases by May 28; **Table 1**) [1-3]. The country’s extensive case detection and care, rigorous contact tracing, and daily monitored mandated quarantines with a people-centered approach have successfully curbed community spread whilst schools and businesses remain open, offering valuable insights for health authorities eager to reinstitute or strengthen their pandemic response while safely reopening their economy.

**Table 1:** COVID-19 case numbers and related measures in Taiwan and selected countries – January 21 to May 26, 2020*

Country (population)	Confirmed case number											Infection rate per million		Mortality rate per million (CFR)	
***% case increase over the past 2 weeks, in italic***										
**(number of deaths)**										
**Date**	**1/21†**	**2/4**	**2/18**	**3/3**	**3/17**		**3/31**	**4/14**	**4/28**	**5/12**	**5/26**	**as of 5/26/2020**			
Taiwan (23.8M)	1	11	22	42	77		322	393	429	440	441	18.5		0.3 (1.6%)	
	· ·	· ·	*100.0%*	*90.9%*	*83.3%*		*318.2%*	*22.0%*	*9.2%*	*2.6%*	*0.2%*				
	(0)	(0)	(1)	(1)	(1)		(5)	(6)	(6)	(7)	(7)				
USA (330.5M)	1	11	13	80		7.0k	186.1k	608.5k	1,027k	1,391k	1,708k	5,167.9	302.9 (5.9%)	
	· ·	· ·	*18.2%*	*515.4%*		*8,650%*	*2,559%*	*227.0%*	*68.8%*	*35.4%*	*22.8%*			
	(0)	(0)	(0)	(9)		(97)	(3.7k)	(24.4k)	(58.9k)	(83.2k)	(100.1k)			
China (1,400M)	400	24.3k	74.2k	80.3k		80.9k	81.6k	82.3k	82.8k	82.9k	83.0k	59.3	3.3 (5.5%)	
	· ·	*5,975%*	*205.3%*	*8.2%*		*0.7%*	*0.9%*	*0.9%*	*0.6%*	*0.1%*	*0.1%*			
	(17)	(490)	(2.0k)	(3.0k)		(3.2k)	(3.3k)	(3.3k)	(4.6k)	(4,6k)	(4.6k)			
Japan (126.0M)	1	19	59	287		873	2.2k	7.9k	13.9k	16.0k	16.6k	131.7	6.8 (5.2%)	
	· ·	· ·	*210.5%*	*386.4%*		*204.1%*	*152.0%*	*259.1%*	*75.9%*	*15.1%*	*3.8%*			
	(0)	(0)	(1)	(6)		(29)	(66)	(146)	(413)	(678)	(860)			
South Korea (51.3M)	1	16	46	4.8k		8.3k	9.8k	10.6k	10.8k	10.9k	11.2k	218.3	5.2 (2.4%)	
	· ·	· ·	*187.5%*	*10,335%*		*72.9%*	*18.1%*	*8.2%*	*1.9%*	*0.9%*	*2.8%*			
	(0)	(0)	(0)	(28)		(81)	(162)	(222)	(244)	(258)	(269)			
Hong Kong (7.4M)	0	18	62	100		167	714	1,012	1,037	1,047	1,065	143.9		0.5 (0.4%)	
	· ·	· ·	*244.4%*	*61.3%*		*67.0%*	*327.5%*	*41.7%*	*2.5%*	*1.0%*	*1.8%*				
	(0)	(1)	(2)	(2)		(4)	(4)	(4)	(4)	(4)	(4)				
Singapore (6.0M)	0	24	81	110		266	926	3.3k	14.9k	24.7k	32.3k	5,383.3		3.8 (0.1%)	
	· ·	· ·	*237.5%*	*35.8%*		*141.8%*	*248.1%*	*256.4%*	*351.5%*	*65.8%*	*30.8%*				
	(0)	(0)	(0)	(0)		(0)	(3)	(10)	(14)	(21)	(23)				
Germany (81.5M)	0	12	16	196		7.2k	61.9k	132.2k	156.3k	170.5k	179.0k	2,196.3		101.8 (4.6%)	
	· ·	· ·	*33.3%*	*1,125%*		*3,573%*	*759.7%*	*113.6%*	*18.2%*	*9.1%*	*5.0%*				
	(0)	(0)	(0)	(0)		(13)	(583)	(3.5k)	(5.9k)	(7.5k)	(8.3k)				
The UK (66.6M)	0	2	9	51		2.0k	25.2k	94.9k	161.1k	226.4k	265.2k	3,982.0		555.6 (14.0%)	
	· ·	· ·	*350.0%*	*466.7%*		*3,822%*	*1,160%*	*276.6%*	*69.8%*	*40.5%*	*17.1%*				
	(0)	(0)	(0)	(0)		(71)	(1.8k)	(12.2k)	(21.7k)	(32.7k)	(37.0k)				
France (65.7M)	0	6	12	212		7.7k	52.1k	131.4k	196.7k	213.7k	219.9k	3,347.0		433.8 (13.0%)	
	· ·	· ·	*100.0%*	*1,667%*		*3,532%*	*576.6%*	*152.2%*	*49.7%*	*8.6%*	*2.9%*				
	(0)	(0)	(1)	(4)		(157)	(3.5k)	(15.7k)	(23.7k)	(27.0k)	(28.5k)				
Italy (60.0M)	0	2	3	2.3k		31.5k	106.8k	162.5k	201.5k	221.2k	230.6k	3,843.3		550.0 (14.3%)	
	· ·	· ·	*50.0%*	*76,567%*		*1,270%*	*239.0%*	*52.2%*	*24.0%*	*9.8%*	*4.2%*				
	(0)	(0)	(0)	(79)		(2.5k)	(12.4k)	(21.1k)	(27.4k)	(30.9k)	(33.0k)				
Spain (46.0M)	0	1	2	119	11.2k		94.4k	174.1k	210.7k	228.0k	236.3k	5,137.0		589.1 (11.5%)	
	· ·	· ·	*100.0%*	*5,850%*	*9,312%*		*742.9%*	*84.4%*	*21.0%*	*8.2%*	*3.6%*				
	(0)	(0)	(0)	(0)	(491)		(8.2k)	(18.3k)	(23.8k)	(26.9k)	(27.1k)				
Australia (25.2M)	0	13	15	35	375		4.6k	6.4k	6.7k	7.0k	7.1k	281.7		4.0 (1.4%)	
	· ·	· ·	*15.4%*	*133.3%*	*971.4%*		*1,127%*	*39.1%*	*4.7%*	*4.5%*	*1.4%*				
	(0)	(0)	(0)	(1)	(5)		(19)	(62)	(84)	(97)	(102)				
World (7,800M)	405	24.6k	75.2k	92.1k	194k		838.6k	1,980k	3,127k	4,293k	5,612k	719.5		44.8 (6.2%)	
	· ·	*5,974%*	*205.7%*	*22.5%*	*110.6%*		*332.3%*	*136.1%*	*57.9%*	*37.3%*	*30.7%*				
	(6)	(492)	(2.0k)	(3.1k)	(7.9k)		(41.4k)	(126.6k)	(216.4k)	(290.5k)	(349.1k)				

M – million, CFR – case fatality rate, calculated as death/confirmed case

*Source: refs. [1,2]. Due to time differences and cut-off hours of each reporting, some case or death numbers may be recorded with slight discrepancies in different reports, news, or publications.

†January 21, 2020 was chosen as the starting date for the comparison table because it was the date both Taiwan and the US confirmed its first, imported case (Japan reported its first case on January 16 and South Korea on January 20).

## SCALING-UP TESTING-TREATMENT CAPACITY AND AUGMENTING DIRECT CARE WORKER SAFETY

Since mid-January 2020, Taiwan Centers for Disease Control (CDC) has developed and implemented systematic testing with <4-hour diagnostic kits and continued to expand testing criteria and capacity, including developing rapid tests for RT-PCR and antibodies. The extensive infectious diseases network includes selected hospitals, health systems, and private clinics, as well as local health departments with rural health centers across the country, each with delineated, coordinated facility emergency responses to meet medical surge needs. The National Health Insurance (NHI) covers >99% of the population and supports risk identification [4,5]. Its easy access and affordability allow those experiencing minor or no symptoms but at risk due to travel or contact histories to readily seek medical attention or get tested; confirmed cases are admitted to isolation rooms for observation and treatments with relatively good results (seven deaths and 95% of cases recovered from COVID-19 thus far) [3], minimizing nosocomial and local transmissions.

To protect the safety and well-being of health care personnel and frontline workers, the Central Epidemic Command Center (CECC) increased reserves of essential medical supplies and personal protective equipment (PPE) from 30 to 45-60 days. Stockpiles are strategically stored at hospitals, CDC, its contracted suppliers, and local health departments and are also available for long-term care and related non-medical facilities and public-facing agencies.

Hospital workers and members of the greater disease prevention network, such as district administrative staff and police officers monitoring quarantine, infected while on duty can apply for government compensation up to US$ 12 000; if they die from COVID-19, their family is eligible for US$ 330 000. CDC frequently re-evaluates PPE standards and precautionary procedures for high-risk workers such as frontline infection control staff and airline crews. Moreover, the Taiwanese public recognizes their critical role. For example, companies and citizens have purchased and delivered lunches to hospitals and CDC, and restaurants and stores offer discounts to health care workers to show their appreciation.

## EXECUTING THOROUGH CONTACT TRACING

In Taiwan, contact tracing is a cross-departmental, human resource-intensive task. Central and regional CDC epidemiologists lead local health department teams in conducting interviews and compiling lists of locations the infected persons have been 7-14 days prior to estimated disease onset and all identifiable contacts, sometimes hundreds per case. Teams work closely with local law enforcement and use data from multiple sources, including matching clinical records from the NHI with travel histories from the Customs and Immigration database. When needed, community security videos and individual cell phone GPS records or social media posts are utilized (with verbal consent) to assist recall, while maintaining confidentiality. Information regarding symptom progression, occupations and travel/contact histories of the infected and suspected, length and proximity of interactions, mask or other precautions employed, and specimen samples are collected to help triangulate the source of infection and determine the risk to contacts.

The first round of case investigation in Taiwan is usually completed within 10 hours, accomplished by teams working extended hours to swiftly halt the spread of transmission to COVID-19 [6]. Every close contact is interviewed by phone or, preferably, in person and tested. If negative, they undergo a 14-day home-quarantine. All other contacts are communicated by telephone and instructed to self-monitor for two weeks. Local environmental departments disinfect identified locations and surrounding areas, as needed. If there is a potential exposure by the larger unidentifiable public, CECC publicizes the site and date through cell broadcast or regular media, to alert affected individuals to also self-monitor. Daily press conferences outlining case investigation results (using case numbers and brief, general descriptions such as “male electrician” or “60-year-old homemaker” to preserve anonymity) have educated the public about transmission routes and underscored the importance of vigilance and cooperation with response efforts.

## ENFORCING QUARANTINE

Taiwan’s low case number could be attributed to a strict yet thoughtful quarantine model, from preemptive health screening by CDC-surveilled airport border control to meticulous contact tracing and self-quarantine, utilizing both incentives and deterrents with wraparound services. Suspected cases self-reported or detected at airports are tested onsite and transferred to hospitals. Passengers arriving from highly affected areas are required to complete a 14-day home-quarantine. Government-subsidized “disease-prevention taxis” and optional, designated hotels are available to avoid potential public or family exposure. Staff from local civil offices visit quarantined individuals upon home arrival to set GPS parameters on their smart phones and provide instruction and a care package. Monitoring consists of electronic location confirmation and one-two phone calls daily to check health status and offer support. A 24-hour hotline provides counseling and information or health care arrangements; staff and volunteers from Quarantine Care Centers offer additional assistance such as planning grocery/meal delivery or childcare. Local environmental protection departments pick up garbage twice weekly to minimize contamination.

**Figure:**
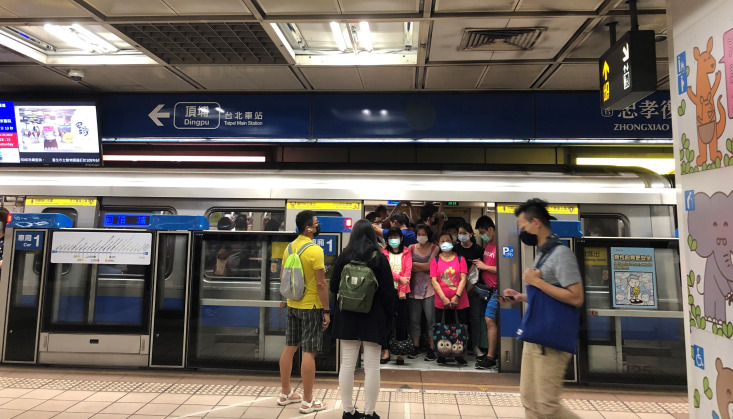
Photo: The metro in Taipei, Taiwan metro (from Cheryl Lin’s collection; used with permission).

To compensate for lost wages while staying home, the government provides a supplement of NT$1000/d for 14 days (nearly US$500) to people lacking paid sick leave or without caregiver days and supporting an ill or quarantined family member [7]. Employers are encouraged to pay these employees and can receive tax credit double the amount paid out. Quarantine violators are located by police officers and may be fined US$3300-33,000, forfeiting financial compensation [8]. Repeat offenders are confined to centralized quarantine facilities for the remainder of their 14-day mandate. As the number quarantined grew, the government implemented a supplementary two-way monitoring chatbot to reduce the human resources burden.

## REINFORCING PUBLIC AWARENESS, SUSTAINING UBIQUITOUS HYGIENE PRACTICES, AND IMPLEMENTING SOCIAL (PHYSICAL) DISTANCING

The emergence of COVID-19 and memories of SARS created a collective sense of urgency early on, prompting proactive, voluntary hygiene measures nationwide: frequent hand washing and sanitizing, mask usage, ubiquitous application of alcohol-based cleaning solutions, and self-observation of symptoms. Temperature checks and hand sanitizer application became standard entryway procedures at schools, hospitals, businesses, apartment complexes, and cultural sites/events starting in late January. Diligently implemented by the majority of Taiwanese, these public health practices helped slow and reduce virus transmission.

Rather than closing schools and businesses or prohibiting private gatherings and public events, CECC provided guidelines, such as remaining 1.5 m apart indoors and 1-m outdoors (otherwise requiring mask-wearing); utilizing plexiglass dividers in classrooms, banks, food courts, and public services; decreasing occupancy of theaters and restaurants; and advance collection of event participants’ contact information for case tracing, if necessary. Although physical gestures of affection between friends and relatives are less customary in Taiwan than in Western countries, close social interactions are common, so the feasibility of social distancing has been under discussion. These guidelines are gradually being relaxed while precautionary measures are maintained.

Anticipating decreased travel restrictions, Taiwan has strengthened community surveillance and preparedness. Policies regarding school and business suspension or city-wide sheltering orders in the event of wide community spread have been reexamined. Some government agencies have implemented provisional dual-team operation, the workforce in each department divided into teams working parallel in different spaces without physical interactions to ensure ongoing operations should an infection cluster occur.

## POLICY IMPLICATIONS

Sustaining a robust public health emergency preparedness and response system that leverages the intelligence of a coordinated infectious disease network has been key to Taiwan’s success. Some of the aggressive pandemic prevention measures, including innovative use of data and technology similarly implemented in South Korea, Australia, the UK, and France [9-11], may raise privacy concerns in other countries [12,13]. Because sophisticated digital tracing or surveillance tools only work when enough people participate in the system, population-based education about the value proposition of these technologies could aid utilization decisions.

Policy outcomes are influenced by people’s trust of government, cultural notions regarding individual civil liberties, and the functionality of the existing system, all of which determine public willingness to support ongoing mitigation efforts as countries reopen. While some of Taiwan’s actions may not be feasible or replicable in other nations [14-16], the principles of advance planning, collective commitment, and thorough execution with a comprehensive, people-centered approach could be useful. Nationally or jurisdictionally, some of the preliminary steps could be implemented with a perspective embracing the hard lessons learned during the first stage of the pandemic, even without a paradigm-shifting policy environment. In Taiwan, early, widely broadcast communication about evolving infection information and policy updates has delivered consistent messages, conveyed transparency, and helped educate the population about self-protection and appropriate behavior. Even in Taiwan, low or decreasing case numbers could induce a false sense of security–prevention communication must stress sustained vigilance and avoidance of complacency before proven treatments or vaccines become available. Strengthening and restoring the linkages between policies, communication, implementation, and public adoption of prevention and response measures remain critical now and into the future as many parts of the world encounter the emergence of new clusters of infections.

Taiwan’s system is not perfect; certain policies, such as the timing of instituting travel advisories and the decision not to perform mass testing of incoming travelers despite popular demand, have invoked criticism. The government has continued to assess, learn from, and refine their pandemic strategies. After larger-than-anticipated crowds congregated in tourist destinations during a 4-day holiday in April, on day three CECC sent out (some thought belatedly) broadcasts recommending that people avoid these sites [17]. Subsequently, a government-commissioned app was soon introduced to inform and manage crowds at popular locations. The public’s enthusiasm to return to “normal” signaled how challenging it is to uphold procedures or reinstall extra measures once guidelines or attention are relaxed or if cases again grow exponentially. When questions arose regarding mask requirements in public buildings, contradicting the Financial Supervisory Commission’s (FSC) rule of removing facial covering when entering banks, FSC issued a temporary injunction to correspond to the disease prevention measure [18]. This discrepancy highlighted the need for policy alignment.

As policy makers around the world tackle the intricate balance between safely reopening the economy and potential resurgence of COVID-19 when schools, businesses, and travel resume, jurisdictions will need to continue pandemic measures including testing, contact tracing, and isolation and consider the conditions necessitating reinstitution of restrictions. Taiwan’s enhanced response demonstrates how a robust system and integrated set of policies including complementary social provisions, along with public compliance, has yielded excellent containment and if more widely adopted, could better defend us all from future threats.
